# A Brown Tumor of Tibial Diaphysis Masquerading as Malignancy

**DOI:** 10.7759/cureus.1319

**Published:** 2017-06-06

**Authors:** Raju Vaishya, Amit Kumar Agarwal, Vipul Vijay, Abhishek Vaish

**Affiliations:** 1 Department of Orthopedics, Indraprastha Apollo Hospital, New Delhi

**Keywords:** cortical lesion, diaphysis, brown tumor, malignancy, tibia

## Abstract

A cortical lesion of the tibial diaphysis in a young patient poses a diagnostic challenge to the clinician. Brown tumors, although not very common, may mimic malignancies on radiographs. Brown tumors are destructive lytic lesions of the bone due to increased osteoclastic activity and fibroblastic proliferation in patients with uncontrolled hyperparathyroidism (HPT). They occur after primary or secondary HPT due to renal failure but very rarely due to vitamin D deficiency. We report a rare case of a brown tumor of tibial diaphyses in a young female patient mimicking a locally aggressive tumor with secondary HPT due to a severe vitamin D deficiency. The effect of hyperparathyroidism on bone metabolism is clinically worse in the presence of vitamin D deficiency and, hence, it predisposes patients to brown tumors that can affect any bone in the body. They can be managed conservatively but may require prophylactic fixation in particular cases.

## Introduction

Brown tumors are focal destructive lesions of the bone caused by increased osteoclastic activity and fibroblastic proliferation in patients with uncontrolled hyperparathyroidism (HPT). They occur after primary or secondary HPT due to renal failure but very rarely due to vitamin D deficiency [1]. The incidence in primary HPT has been found to be around 4.5% while it is around 1.5-1.7% in secondary HPT [2]. The brown tumor consists of fluid-filled cysts rich in highly vascularized fibrous tissue containing hemorrhagic spots. Their brown color is because of increased hemorrhage and hemosiderin deposition [3]. These can be located in any part of the skeleton, but are most frequently encountered in the ribs, clavicles, ends of the extremities, and pelvic girdle. Clinically significant lesions in the diaphysis of the tibia are rare and pose a diagnostic challenge to the clinician. We report a rare case of brown tumor of tibial diaphyses mimicking a locally aggressive tumor in a female with secondary HPT due to severe vitamin D deficiency.

## Case presentation

**Figure 1:**
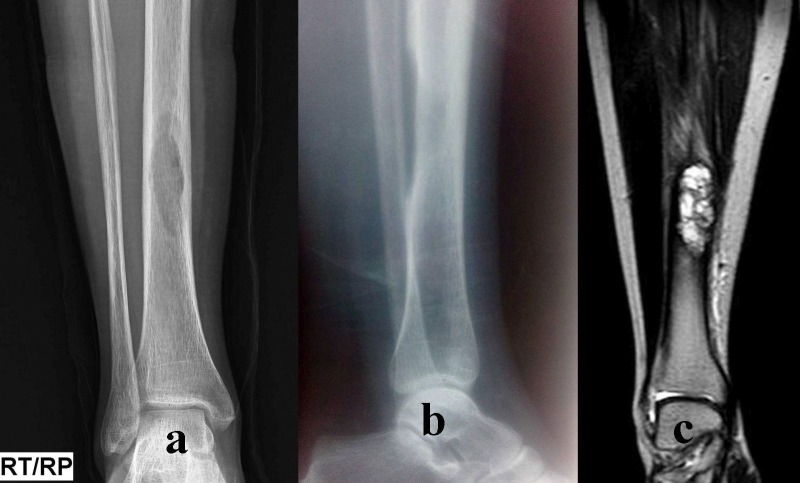
Figure 1(a) anteroposterior, 1(b) lateral views of brown tumor of right tibial diaphysis in 25-year-old female patient. 1(c) magnetic resonance imaging (MRI) showing well-defined expansile lytic lesion along the anterior medial aspect of the middle third of shaft of the right tibia. Hyperintense on T2 and iso to hypointense on T1.

A 25-years-old breastfeeding patient, after nine months of delivery, presented with right leg pain associated with difficulty in walking for the past one month. She had tenderness over the posteromedial aspect of the right distal third leg. Plain radiographs showed a geographical lytic lesion in the diaphysis of the distal one-third of the tibia with complete erosion of the posteromedial cortex (Figure 1).

Her blood parameters were deranged as shown in the table below (Table 1). Her vitamin D values were very low and serum parathyroid hormone (PTH) levels were very high. Skeletal survey was done to rule out any other lesion.

**Table 1:** Laboratory parameters at the time of admission and at the end of three months.

Investigations	Normal Range	Initial Values	Values at Three Months
Parathyroid hormone (PTH), pg/ml	15-65	495	60
Calcium, mg/dl	8.8-11	9.0	8.9
Phosphorus, mg/dl	2.5-5.0	4.3	4.5
Alkaline phosphatase, IU/L	65-300	450	280
25(OH) Vitamin D, ng/dl	>30.0	<3	32.0

Based on the history, clinical examination, and laboratory tests, the final diagnosis of brown tumor was made secondary to severe Vitamin D deficiency induced secondary HPT. The differential diagnosis of the cortical diaphyseal lesion of the tibia is described in the table below (Table 2).

**Table 2:** The differential diagnosis for a cortical lesion of the tibia.

		Pathology	Clinical features	Radiological features
1	Brown tumor	Increased osteoclastic activity and fibroblastic proliferation in uncontrolled hyperparathyroidism	Occurs at all ages, more commonly seen in females	Osteolytic geographical lesions with sharp margins
2	Osteofibrous dysplasia	It is considered a fibrovascular defect and demonstrates unique osteoblastic rimming and bone zonation	Patients usually of the 1st or 2nd decade, bowing and enlargement of the bone are seen.	The middle to distal third of the diaphysis with a predilection for the anterior cortex.
3	Eosinophilic granuloma	Non-neoplastic proliferation of histiocytes	Patients present at less than 20 years. The male-to-female ratio is usually 2:1.	A centric lesion, the appearance consists of a lytic lesion with variable bone destruction; the lesion may appear aggressive.
4	Ewing sarcoma	It is a highly malignant small, round, blue cell tumor	The patient is usually under 30 years; the male-to-female ratio is 3:2.	Permeative lesion that often elicits multilayered periostitis
5	Adamantinoma	It is a rare, locally aggressive lesion	The patient is usually 20–50 years. The male-to-female ratio is 1.3:1.	Anterior cortex and the middle third is usually involved; epicenter is typically eccentric. Multilocular or slightly expansile osteolytic lesion, which may be locally aggressive
6	Hemangioendothelioma	It is a malignant bone tumor; the lesion is composed of irregular, anastomosing vascular channels	30–50 years. The male-to-female ratio is usually 2:1.	Centric or eccentric epicenter. The appearance consists of lytic, often multiple lesions, characteristically in a regional distribution
7	Fibrous dysplasia	Benign disorder characterized by tumor-like proliferation of fibro-osseous tissue	Age is typically 20–30 years, the male-to-female ratio is usually 1:1	Ground-glass lucency with irregular but well-defined borders, and a mildly expansile lesion

**Figure 2:**
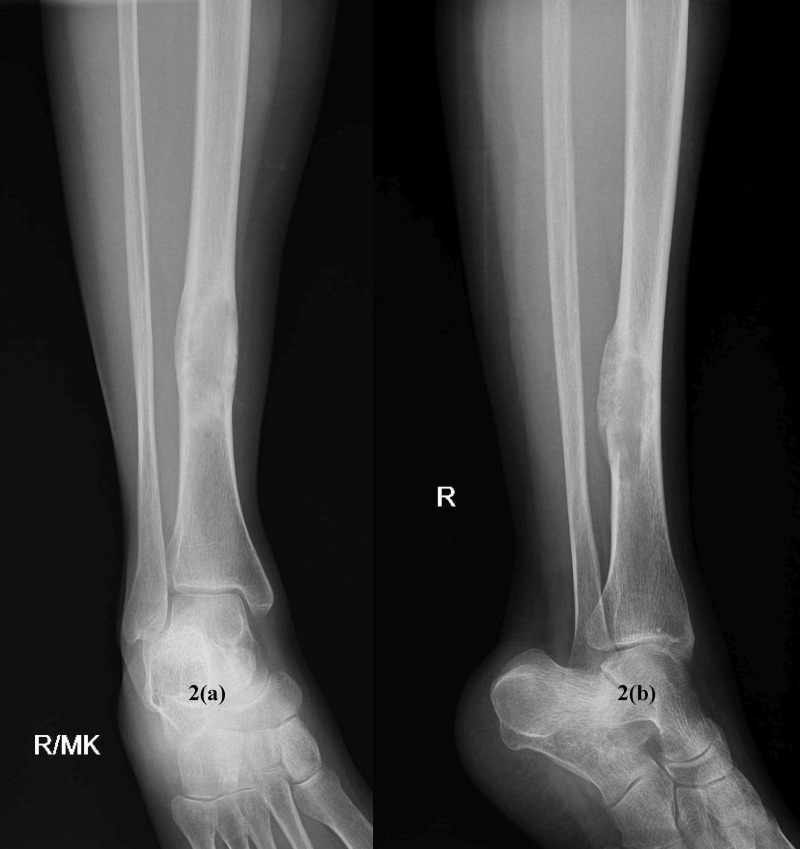
2(a) anteroposterior and 2(b) lateral view of healed lesion of right tibial diaphysis after treatment with vitamin D supplements.

The patient was treated with vitamin D supplementation, and the leg was immobilized in a below knee cast for two months. Gradually, the tumor regressed. The patient reported clinical improvement, and the biochemical markers came to normal values after three months. Her follow-up radiograph also showed remarkable improvement (Figure 2).

## Discussion

A cortical lesion of the tibial diaphysis in a young patient poses a diagnostic challenge to the clinician.The parathyroid glands play a major role in maintaining extracellular calcium concentrations through the secretion of PTH. Excessive PTH causes the rapid absorption of calcium from the bones, with resultant hypercalcemia in the extracellular fluid. PTH activates osteoblasts and osteocytes, which in turn send signals to osteoclasts, causing them to eat up the bone in an accelerated manner. Osteoclastic resorption of the bone results in weakened bones after a few months of excess PTH. There is secondary stimulation of the osteoblasts that attempt to correct the weak state. In this stage, there is more bone absorption than bone deposition in the presence of continued excess PTH. Prolonged secretion of PTH over a period of many months or years results in very visible absorption in all the bones and the development of large cavities filled with giant multinucleate osteoclasts. The large cavities act as stress risers predisposing the affected bone to pathological fractures. Vitamin D promotes bone calcification by increasing calcium and phosphate absorption from the intestines. Vitamin D, by an unknown mechanism, also causes the transport of calcium ions through cell membranes, but in this instance, in the opposite direction to the osteoblastic or osteoclastic cell membrane. Therefore, the effect of hyperparathyroidism on bone metabolism is clinically worse in the presence of vitamin D deficiency and hence predisposes patients to brown tumors that can affect any bone in the body.

The main effects of PTH are to increase the level of plasma calcium by increasing the release of calcium and phosphate from the bone matrix, increasing calcium reabsorption by the kidneys, and increasing renal production of 1,25-dihydroxy vitamin D3 (calcitriol), which in turn increases intestinal absorption of calcium. Primary hyperparathyroidism is a well-recognized entity identified almost more than a century ago by Von Recklinghausen. In the USA, the annual incidence is about 0.2% in patients above the age of 60 years. This condition is more common in females than in males, with the peak incidence between 30 to 50 years. The incidence increases with age, though a 14-year-old patient has been reported in the literature.

The discussion points, therefore, include the role of Vitamin D supplementation, the role of partial parathyroidectomy, and the role of prophylactic internal fixation of the tibia as the most relevant treatment options.

Calcium and vitamin D supplementation have been found very useful in the treatment of brown tumors of the spine following spine decompression for collapsed vertebrae. Though surgical options are helpful in the treatment of pressure symptoms associated with a brown tumor, the pharmacological option still plays an additive role in the management of brown tumors. Surgical removal of three of the four normal parathyroid glands causes transient hypoparathyroidism. A few studies have reported improvements in clinical outcomes among patients with brown tumor treated with parathyroidectomy. The return of serum biochemistry to normal, increase of bone density, and fracture union following excision of parathyroid adenoma have been reported. The prophylactic internal fixation for large bone lesions should be done to prevent spontaneous fracture [4]. Significant clinical improvement has been found among patients with bone defects treated with tumor curettage and stabilization of the long bone with less invasive stabilization systems [5]. It must be supplemented with calcium and vitamin D. Therefore, surgical stabilization of a complete or impending pathological fracture should be accompanied by calcium and vitamin D supplementation to restore normal body biochemistry.

## Conclusions

Brown tumors may mimic malignancies on radiographs. Lesions in the diaphysis of the tibia are rare and pose a diagnostic challenge to the clinician. Brown tumor should be considered as one of the differential diagnosis in tibial diaphyses lesions. It can present as a locally aggressive tumor in a patient with secondary HPT due to a severe vitamin D deficiency. Medical management is sufficient in most of the cases; however, prophylactic surgical fixation may be required in selected cases.
